# 

**Published:** 1855-01

**Authors:** 


### INDEX TO VOL. II.

Advice to Housekeepers......... 23
A suggestion to Christians........ 135
A Life saving Thought.......... 156
Alcohol a Poison................ 225
Appetite, What is it............. 237
Agriculture Elevating........... 260
Arguing Successfully..........35, 281
An experiment certified.......... 283

Bible and Materia Medica........ 40
Bad Temper and Insanity........ 118
Bites and Stings Cured........... 160
Bread Making.... ...........147, 271
Be constantly employed.......... 157
Be Systematic................. 231
Be Careful..................... 242
Be Courteous.................. 255
Be Kind....................... 256

Cough Medicines................ 4
Cleaveland on the Skin........... 36
Charcoal Fumes, Poison.......... 74
Cleaveland on Food, and Drink... 88
Common Colds............50, 53, 116
Constitution Hardening.......... 119
Cockroach Riddance.............. 122
Clergymen Dyspeptic..........33, 135
Cabbage Culture................ 152
Cholera, Essay on..... ......17, 169
Church Music............. ...65, 234
Census Items.................. 235
Consumption Described.......168, 236
College of Cookery.............. 245
Costiveness..................25, 267
Cold Feet.................8, 35, 277
Children............68, 161, 231, 279
Climate Changing............93, 280
Clothing Changing Critical...284, 286

Debt and its Results............. 60
Don't get Discouraged........... 90
Death's Doings...............49, 91
Drowning..................... 104
Dieting for Health.............. 117
Deaf and Dumb.... ............ 123
Diet in Cholera times............ 190
Decision of Character............ 238

Domestic Receipts...........124, 244
Dyspeptia...................31, 257
Doctors' Value..............233, 262
Duty of the Elevated to the Lowly. 284

Early Rising.................... 81
Eyes, Management of.........71, 114
Eloquence, True................. 145
Eating too much..............30, 241
Emotions and Digestion.......... 282

Fruits and their use............. 153
Fire Kindling................... 258
Food the best Physic......88, 147, 267
Finger Nails, Management........ 276
Feet.....................8, 55, 277

Going in Debt.................. 60
Green, Dr. Horace tribute to...... 163
Grapes, and how to use them..... 268

How to Live Long.............. 1
How Persons become Costive..... 5
How to be Healthy............. 15
How to Consult a Physician....... 27
How much must I Eat........... 30
How People take Cold........... 50
How to Cure a Cold............. 53
How to harden the Constitution... 119
How to Preach effectively..... .. 121
How to leave Church in Winter... 140
How to be Eloquent............. 145
How to be Happy.............8, 231
Heartburn what is it............ 29
Health and Hard Study.......... 31
Hall, Samuel Wallace obituary.161, 176
Hall, Caroline Amelia, Poetry of.. 78
Houses, health of locality, &c..... 105
Hominy and its use...........71, 149
Homœopathy in Cholera Times... 217
Harper, James, Tribute to........ 256
Hamilton, Mrs. Alexander........ 256
Hereditary Disease.............. 268
Help the Lowly................. 284

Inhalation for Consump. 159, 219, 247
Insanity................118, 124, 260

Illnatured People.................. 282
Kindling Fire.................... 258

Long Life, how Secured.......... 1
Lozenges, effect of............... 3
Lesson to Parents................ 116
Long Prayers.................... 121
Life saving Thoughts............ 156
Lasyrites, Essay on...........25, 254

Milk Sickness.................... 57
Milch Cows in Winter........... 64
Morning Walks Mischievous...... 82
Mental Epidemics................ 94
Mind and Health................. 111
Measurement Rules.............. 124
Money a Medicine............... 156
Music in Churches.........65, 70, 234
Medical Progress................ 240
Medicated Inhalation....159, 219, 247
Mothers......................... 279

Nursing the Sick................ 12
Night Air...................... 83
Natural Death.................. 91
Newspapers, Conduct of..165, 249, 252

Ousting from Pews, prevented.... 143
Old Age Statistics.......21, 50, 67, 233
Owls and their wisdom ......... 234

Poetry....................20, 44, 78
Popular Fallacies................ 81
Peter Wife's Mother............. 120
Pew System in Churches......... 143
Preaching Effectively.....21, 139, 145
Poisons, and their Cure,....59, 74, 224
Physicians Life Time............ 233
Progress of our Principles........ 296

Plea for Poor Children........... 271
Poverty and its Elevation.53, 239, 285

Regularity of Life................ 21
Rules for the Year............... 73
Rat Riddance.................... 122
Regaining Health................ 136

Skin, Prof. Cleaveland on........ 35
Solar Heat...................... 72
Sudden Death................... 75
Spiritualism...................94, 125
Suggestions to the Church........ 135
Summer Complaint prevented..... 184
Sleeping well................241, 279
Statistics Measurement........... 124
" nourishment of Food.......... 151
" Old Age...........21, 50, 97, 233
" Census...................... 235
" Consumption................. 236
Shoes for Winter................ 287

To live Long.................... 1
Teachings of Bible on Health..... 40
Transplanting Trees............. 69
Tea and its preparations......... 75
To Travel safely................ 87
Temper and Insanity............ 118
Tables of Food valuable.......... 151
Temperance Question............ 233
Throat Ail...................25, 254
True Courage................... 270

Victoria's Children's Habits...... 269
Ventilation..................... 286

Water on the Brain............. 4
"We'll meet Again"............. 78
Wives, different kinds of...23, 68, 263

